# The ATR inhibitor berzosertib acts as a radio- and chemosensitizer in head and neck squamous cell carcinoma cell lines

**DOI:** 10.1007/s10637-023-01408-w

**Published:** 2023-11-07

**Authors:** Julia Schnoell, Carmen Sparr, Sega Al-Gboore, Markus Haas, Faris F. Brkic, Lorenz Kadletz-Wanke, Gregor Heiduschka, Bernhard J. Jank

**Affiliations:** Department of Otorhinolaryngology, Head and Neck Surgery, Medical University of Vienna, Vienna, Austria

**Keywords:** HNSCC, Radiation, Cisplatin, VX-970, VE-822, M6620

## Abstract

Alterations in the DNA damage response play a crucial role in radio- and chemoresistance of neoplastic cells. Activation of the Ataxia telangiectasia and Rad3-related (ATR) pathway is an important DNA damage response mechanism in head and neck squamous cell carcinoma (HNSCC). Berzosertib, a selective ATR inhibitor, shows promising radio- and chemosensitizing effects in preclinical studies and is well tolerated in clinical studies. The aim of this study was to elucidate the effect of berzosertib treatment in combination with radiation and cisplatin in HNSCC. The HNSCC cell lines Cal-27 and FaDu were treated with berzosertib alone and in combination with radiation or cisplatin. Cell viability and clonogenic survival were evaluated. The effect of combination treatment was evaluated with the SynergyFinder or combination index. Apoptosis was assessed via measurement of caspase 3/7 activation and migration was evaluated using a wound healing assay. Berzosertib treatment decreased cell viability in a dose-dependent manner and increased apoptosis. The IC_50_ of berzosertib treatment after 72 h was 0.25–0.29 µM. Combination with irradiation treatment led to a synergistic increase in radiosensitivity and a synergistic or additive decrease in colony formation. The combination of berzosertib and cisplatin decreased cell viability in a synergistic manner. Additionally, berzosertib inhibited migration at high doses. Berzosertib displays a cytotoxic effect in HNSCC at clinically relevant doses. Further evaluation of combination treatment with irradiation and cisplatin is strongly recommended in HNSCC patients as it may hold the potential to overcome treatment resistance, reduce treatment doses and thus mitigate adverse events.

## Introduction

Head and neck squamous cell carcinoma (HNSCC) are among the ten most common cancers. In 2020, HNSCC comprised 4.6% of all new cancer diagnoses and 4.5% of cancer deaths worldwide [1]. Most patients present with locally advanced stages and frequently require multimodal therapy, which includes surgery and radio(chemo)therapy or radiochemotherapy alone [2, 3]. However, treatment with high-dose cisplatin is only possible in nonelderly patients with no major comorbidities [4]. Thus, new therapeutic options are needed to improve the response to radio- and chemotherapy, which could ideally help to reduce their doses and adverse effects.

Ataxia telangiectasia and Rad3-related (ATR) plays a major role in DNA damage response and triggers the S and G2/M cell cycle checkpoints. ATR is activated by DNA single-strand breaks and replication stress, which are both induced by radio- and chemotherapy. HNSCC commonly exhibits an impaired G1/S checkpoint, due to mutation or inactivation of TP53, which is associated with radio- and chemoresistance. Thus, HNSCC cells are dependent on S and G2/M checkpoints. The activation of these checkpoints leads to the arrest of the cell cycle and allows for DNA damage repair. As a consequence, inhibition of ATR (in the G1 checkpoint impaired state) leads to synthetic lethality, while normal adjacent tissue with an intact G1 checkpoint is spared [2].

Berzosertib (also known as VX-970, VE-822 or M6620) is a selective inhibitor of ATR. Treatment with berzosertib as single agent treatment and in combination with chemotherapy and radiotherapy showed promising preclinical anticancer effects in several cancers in vitro [5]. No relevant effect was observed on normal fibroblasts or breast epithelial cells [6–9]. Berzosertib is currently under clinical investigation in several phase I-III studies [10]. First results show that berzosertib is well tolerated as monotherapy and in combination with cisplatin, gemcitabine or topotecan [11–15].

This study aimed to investigate the effect of berzosertib in HNSCC cell lines and the interaction between berzosertib and radiation or cisplatin treatment.

## Methods

### Reagents

The ATR inhibitor berzosertib was purchased from Selleck Chemicals (Houston, TX, USA). A stock solution was dissolved in DMSO at a concentration of 50mM and stored at -20 °C. The chemotherapeutic agent cisplatin (1 mg/ml) was retrieved from ready-to-use infusions from the pharmacy of the General Hospital of Vienna. The working concentration of each agent was prepared directly before each experiment.

### Cell lines and Cell Culture

To investigate the effect of berzosertib treatment the HNSCC cell lines FaDu and Cal-27 (German Collection of Microorganisms and Cell Cultures GmbH, DSMZ, Braunschweig, Germany) were used. Cells were kept at 37 °C and 5% CO_2_ in a humidified incubator. The used cell culture medium was Dulbecco’s Modified Eagle’s Medium (DMEM; Thermo Fisher Scientific, Gibco, Waltham, MA, USA) supplemented with 10% Fetal Bovine Serum (FBS; Thermo Fisher Scientific), 100 U/mL Penicillin, and 100 µg/mL Streptomycin (Thermo Fisher Scientific), further referred to as culture medium. Subculturing was performed at 80–90% confluency and to a maximum passage number of 25.

### Radiation treatment

To investigate the combined effect of berzosertib and irradiation, treatment was combined with 2–8 Gy directly after berzosertib treatment. Cells were irradiated at a dose rate of 1 Gy/min with the 200 kV YXLON Maxishot unit YXLON International GmbH, Hamburg, Germany) as previously described [16]. In detail, irradiation was performed at a distance of 45.5 cm, with a focus size of 5.5 cm and a current of 20mA. A 4 mm aluminum and 0.6 mm copper filter were applied.

### Cell viability assay

To investigate the effect of treatment on the HNSCC cells, a resazurin assay was performed in 96-well plates. Five thousand cells were seeded per well. One day after seeding, cells were treated with berzosertib alone (0.031-1 µM), or a combination of berzosertib (0.016-0.5 µM) and cisplatin (0.31-10 µM) or irradiation (0–8 Gy). 0.1% DMSO was used as a control. After 72 h of incubation, the medium was removed and cells were incubated with medium containing 56 µM resazurin (Sigma-Aldrich, St. Louis, MO, USA). After 2-3 h of incubation, absorbance was measured using a TECAN SPARK 10 M microplate reader (Männedorf, Switzerland) at 570 nm.

### Colony formation assay

Colony formation of Cal-27 and FaDu was measured using the colony formation assay as described by Franken et al. [17]. Cal-27 cells were seeded at a density of 250, 250, 500 and 1000 cells per well for 0, 2, 4 and 6 Gy, respectively. FaDu cells were seeded at a density of 300, 300, 600, 1200 cells per well for 0, 2, 4 and 6 Gy, respectively. After one day of incubation, cells were treated with berzosertib (0.016–0.125 µM) and radiation (0–6 Gy). After 72 h, berzosertib-containing medium was replaced with normal medium and cells were incubated for another ten days. Colony formation was then scanned using a TECAN SPARK 10 M microplate reader and evaluated with ImageJ 1.53e as previously described [18]. Briefly, a binary contrast enhanced image was created and the colonies containing more than 50 cells were counted using the “analyze particles” function. The average pixel area of 50–60 cells was measured and the cut-off for the minimum particle size was adjusted accordingly.

### Apoptosis assay

Apoptosis was measured using the Caspase-Glo® 3/7 assay (Promega Corporation, Madison, WI, USA) according to the manufacturers protocol. The experiment was carried out in a 96-well plate with 5000 cells per well. After 24 h of incubation after seeding, cells were treated with berzosertib alone (Cal-27: 0.25 µM, FaDu: 0.5 µM), and in combination with cisplatin (Cal-27 2.5 µM, FaDu: 5 µM) or radiation (4 Gy) for 48 h. Then, the tissue plate was cooled to room temperature and incubated with the Caspase-Glo® 3/7 reagent for 30 min. The resulting luminescence was measured using the TECAN SPARK 10 M reader. Luminescence levels were normalized to the DMSO 0.1% control.

### Migration assay

Cell migration after berzosertib treatment was measured using 2 chamber culture-inserts (ibidi GmbH, Graefeling, Germany). Cal-27 and FaDu cells were seeded at a density of 60,000 and 70,000 cells per culture-insert in a 24-well plate. After 24 h of incubation, the inserts were carefully removed and cells were treated with berzosertib (0.125-0.5 µM). Gap closure was measured at 0, 24 and 48 h (only for FaDu cells) using the TECAN SPARK 10 M reader and evaluated using the MRI wound healing tool [19] in ImageJ 1.53e.

### Statistical analysis

Data was normalized to the DMSO 0.1% control group. Results were visualized and evaluated using a one-way or two-way ANOVA and Dunnett’s multiple comparisons test in Graph Pad Prism 8 (Version 8.4.2, GraphPad Software Inc., San Diego, CA, USA). The inhibitor’s IC_50_ values were calculated using a nonlinear regression model with a variable slope. Combination experiments with irradiation were evaluated using the SynergyFinder 3.0 and the Zero Interaction Potency (ZIP) method [20, 21]. A ZIP score below -10 indicates antagonism, above 10 synergy and in between an additive effect. Combination experiments with cisplatin were evaluated using the Combination Index (CI) as published by Chou et al. using CompuSyn software (ComboSyn Inc.) [22]. A CI below 0.9 indicates a synergistic effect, between 0.9 and 1.1 an additive effect and above 1.1 an antagonistic effect. A p-value below 0.05 was considered statistically significant.

## Results

### Berzosertib decreases cell viability

**Fig. 1:**
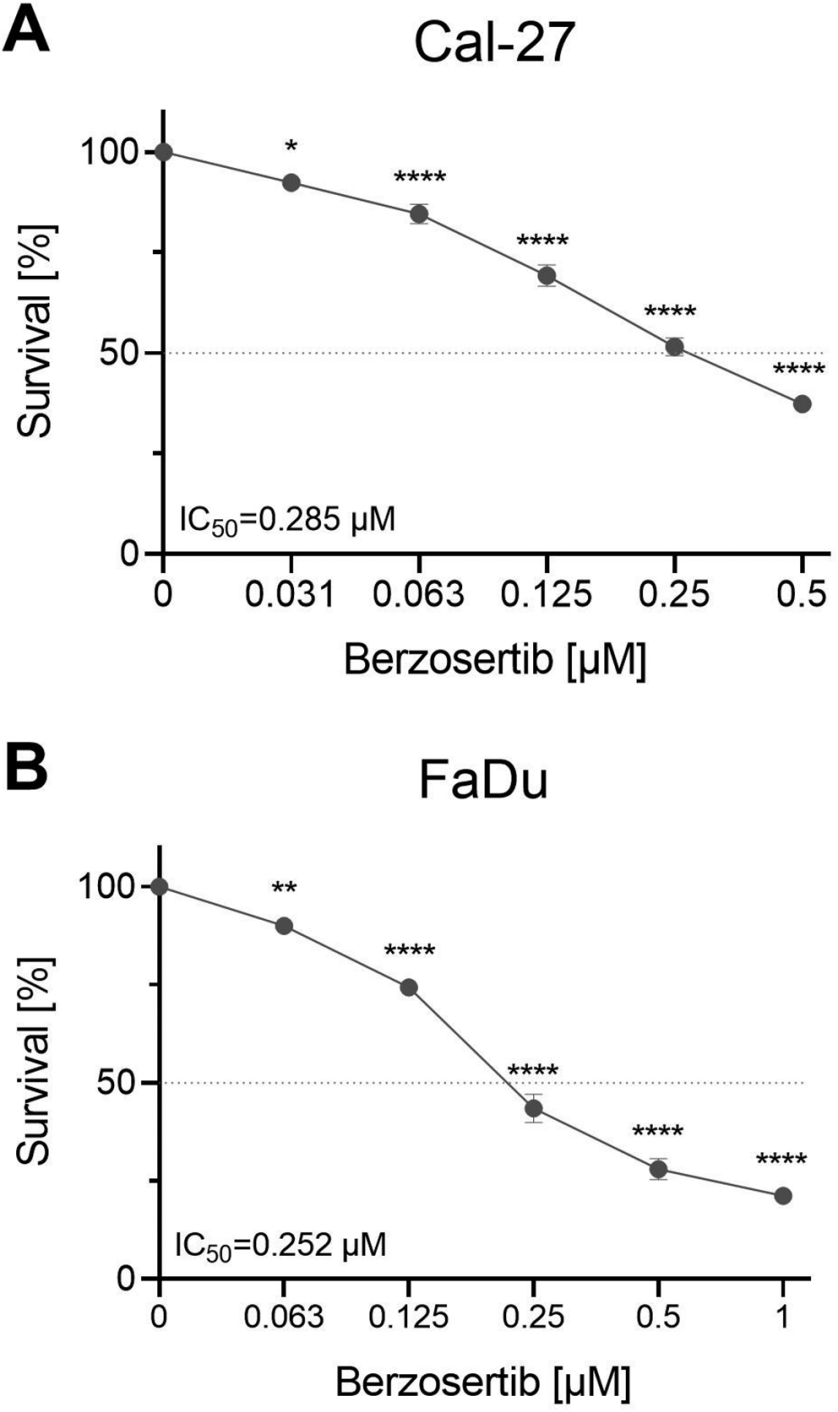
Cell viability assay of berzosertib. Cal-27 (**A**) and FaDu (**B**) cells were treated with berzosertib for 72 h. Cell viability was measured using resazurin. Data were normalized to the untreated (DMSO 0.1%) control group. Error bars represent the standard error of the mean

To investigate the effect of berzosertib on HNSCC cell lines, a cell viability assay using resazurin was performed. The cell lines Cal-27 and FaDu were incubated with berzosertib for 72 h and showed a dose-dependent decrease in cell viability (Fig. 1). The IC_50_ values were 0.285 µM (95%CI: 0.259–0.315) for Cal-27 and 0.252 µM (95%CI: 0.231–0.275) for FaDu.

### Berzosertib acts as a radiosensitizer

**Fig. 2:**
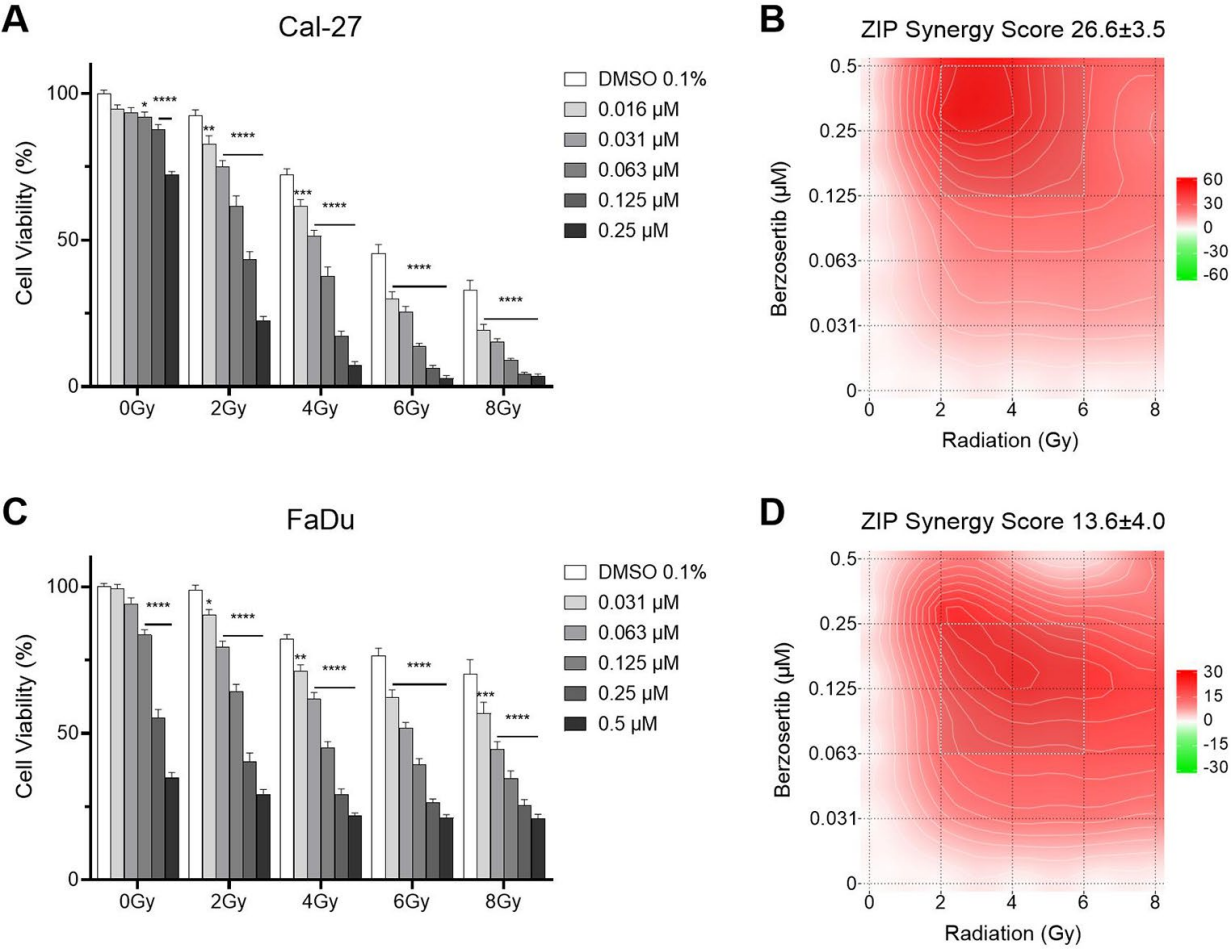
Cell viability assay of berzosertib and radiation. Cal-27 (**A**) and FaDu (**C**) cells were treated with berzosertib and/or radiation for 72 h. Results were evaluated with the synergy finder and zero interaction potency (ZIP) score (**B**,**D**). Data were normalized to the untreated (DMSO 0.1%, 0 Gy) control group. Asterisks represent a significant difference of berzosertib treatment compared to the DMSO control treated with the same radiation dose (*: p < 0.05; **p < 0.01; ***: p < 0.001; ****: p < 0.0001). Error bars represent the standard error of the mean. A ZIP score above 10 indicates synergism, below -10 antagonism, and in between (-10–10) an additive interaction

Combination experiments were carried out using a resazurin assay and a clonogenic assay. After 72 h of incubation, the resazurin assay revealed significant inhibition of cell viability for the combination of berzosertib and irradiation in Cal-27 (p ≤ 0.002) and FaDu cells (p ≤ 0.033, Fig. 2). In detail, 4 Gy of radiation reduced cell viability to 72%, 0.25 µM berzosertib to 72% and combination treatment reduced cell viability to 7% in Cal-27 cells (p < 0.0001). In FaDu cells, irradiation with 4 Gy resulted in a decrease in cell viability to 82%, treatment with 0.25 µM berzosertib to 55% and combination treatment reduced cell viability to 22% (p < 0.0001). Evaluation of the combinatory effect using the SynergyFinder [20, 21] revealed a synergistic effect with an overall ZIP synergy score of 26.6 and 13.6 for Cal-27 and FaDu, respectively (Fig. 2). The most potent doses of the combination were between 0.125 and 0.5 µM and 2–6 Gy for Cal-27 and 0.063–0.25 µM and 2–6 Gy for FaDu.

**Fig. 3:**
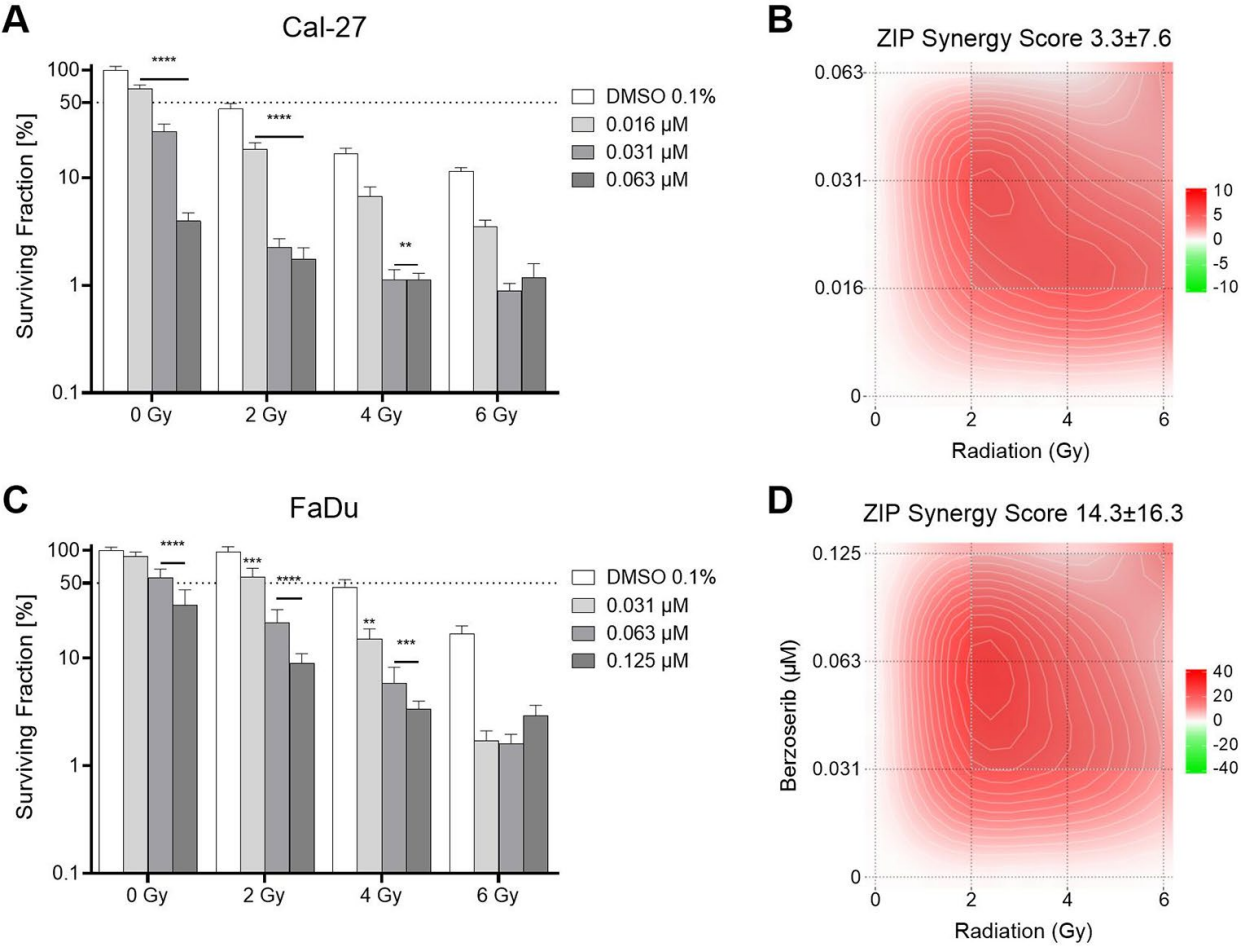
Colony formation assay of berzosertib and radiation. Cal-27 (**A**) and FaDu (**C**) cells were treated with berzosertib and/or radiation and then incubated for ten days. Results were evaluated with the synergy finder and zero interaction potency (ZIP) score (**B**,**D**). Data were normalized to the untreated (DMSO 0.1%, 0 Gy) control group. Asterisks represent a significant difference of berzosertib treatment compared to the DMSO control treated with the same radiation dose (*: p < 0.05; **p < 0.01; ***: p < 0.001; ****: p < 0.0001). Error bars represent the standard error of the mean. A ZIP score above 10 indicates synergism, below -10 antagonism, and in between (-10–10) an additive interaction

To further elucidate the effect on colony formation, the effect of berzosertib in combination with radiation was examined in a clonogenic assay. Again, the combination of berzosertib (0.016–0.125 µM) and radiation (2–4 Gy) exhibited a decrease in colony formation greater than single agent therapy (Fig. 3). In Cal-27 cells, treatment with 2 Gy of irradiation led to a decrease in colony formation to 43%, while treatment with 0.016 µM berzosertib decreased colony formation to 67%. Combination treatment further reduced colony formation to 18% (p < 0.0001). The colony formation of FaDu cells was not affected by 2 Gy. Berzosertib treatment with 0.031 µM alone slightly decreased colony formation to 89% (not significant). However, combination of both treatments led to a decrease in colony formation to 57% (p = 0.0003). The SynergyFinder [20, 21] revealed an additive effect in Cal-27 (ZIP Synergy Score 3.3; Fig. 3B), and a synergistic effect in FaDu cells (ZIP Synergy Score 14.3; Fig. 3D).

### Combination of berzosertib and cisplatin shows a synergistic effect

**Fig. 4:**
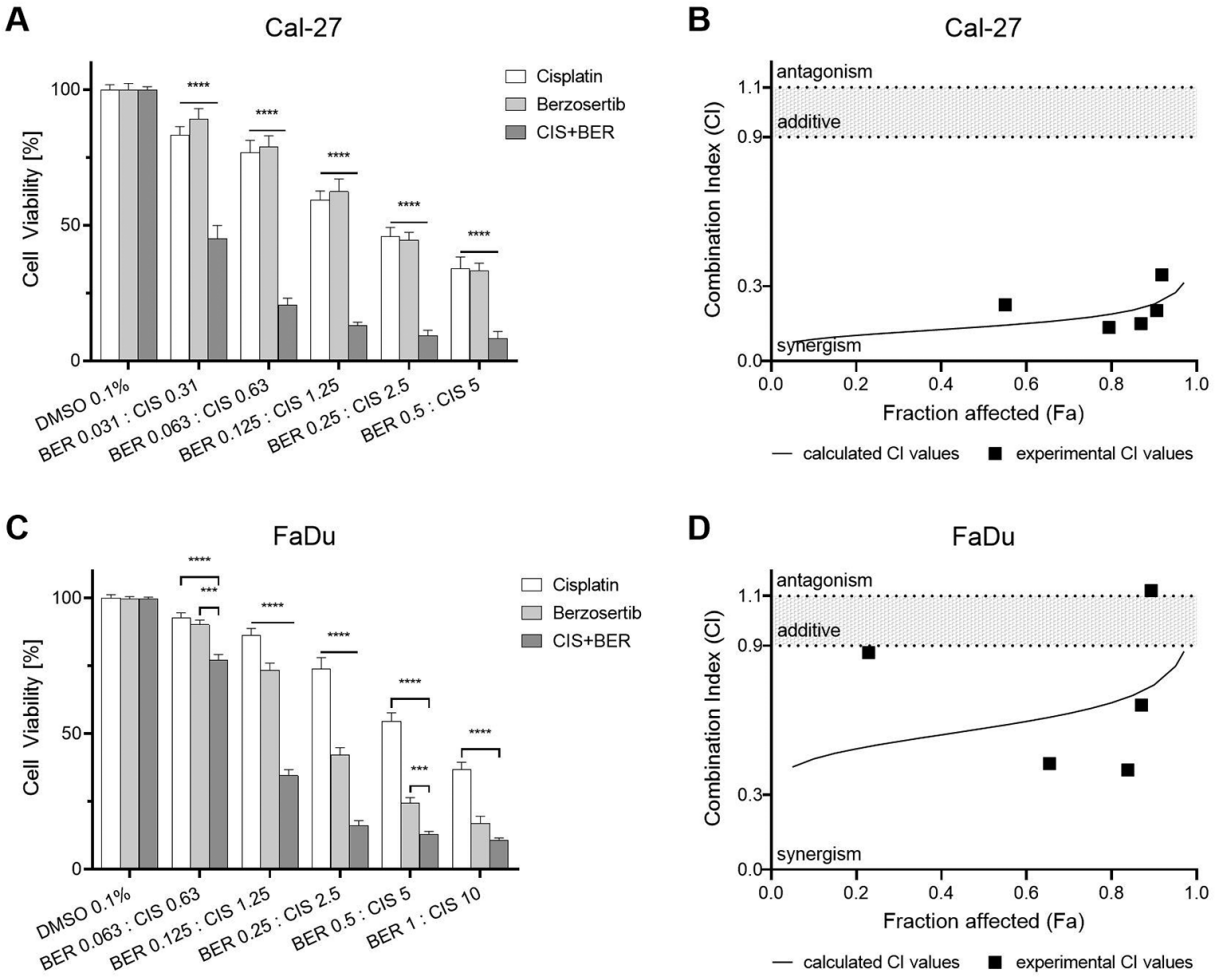
Cell viability assay of berzosertib and cisplatin. Cal-27 (**A**) and FaDu (**C**) cells were treated with berzosertib and/or cisplatin for 72 h. Results were evaluated with the combination index (**B**,**D**). Data were normalized to the untreated (DMSO 0.1%) control group. Asterisks represent a significant difference of combination treatment compared to single agent treatment (*: p < 0.05; **p < 0.01; ***: p < 0.001; ****: p < 0.0001). Error bars represent the standard error of the mean

The combination of berzosertib and cisplatin was evaluated after 72 h of treatment using a resazurin assay and the combination index (CI; Fig. 4) [22]. The combination ratio was chosen using the IC_50_ values of monotherapy as guidance. IC_50_ values of cisplatin treatment were 2.15 µM and 6.08 µM in Cal-27 and FaDu cells, respectively. Thus, a ratio of 1:10 was chosen for combination experiments. In Cal-27 cells, all evaluated doses of combination treatment exhibited a significant reduction in cell viability for the combination treatment when compared to single-agent therapy (all p < 0.0001). Single agent treatment with 2.5 µM cisplatin reduced cell viability to 46% and 0.25 µM berzosertib reduced cell viability to 45%. The combination of both agents at the same concentrations decreased cell viability to 9% (p < 0.0001). Evaluation of Cal-27 cell viability with the CI revealed a strong or very strong synergism (experimental and calculated CI < 0.3) for a dose up to 2.5 µM cisplatin and 0.25 µM berzosertib (fraction affected below 0.97). The combination of 5 µM cisplatin and 0.5 µM berzosertib revealed a moderate synergistic effect (CI: 0.35).

In FaDu cells, the cell viability was decreased significantly up to the combination of 0.5 µM berzosertib and 5 µM cisplatin (all p < 0.001). In detail, 2.5 µM cisplatin decreased cell viability to 74% and 0.25 µM berzosertib to 42%. Combination treatment further reduced cell viability to 16% (p < 0.0001). FaDu cells responded to combination treatment in a nearly additive and synergistic manner. The calculated CI values all indicated a synergistic effect (CI: 0.42–0.88). The experimental CI values revealed a slight synergism for the combination of 0.625 µM cisplatin and 0.0625 µM berzosertib (CI: 0.87), a moderate synergistic effect for the combination of 1.25-5 µM cisplatin and 0.125-0.5 µM berzosertib (CI: 0.40–0.66), and a slight antagonism for combination of 10 µM cisplatin and 1 µM berzosertib (CI: 1.12).

### Berzosertib treatment induces apoptosis

**Fig. 5:**
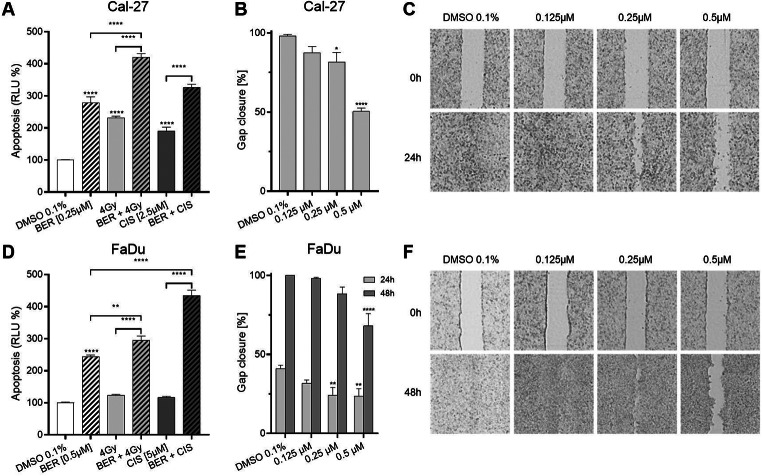
Apoptosis and migration assay. For the apoptosis assay, Cal-27 (**A**) and FaDu (**D**) cells were treated with berzosertib, radiation and/or cisplatin for 48 h and caspase 3/7 activity was measured. Migration was evaluated after 24 h for Cal-27 (**B**,**C**) or 24 and 48 h for FaDu (**E**,**F**) cells. Data were normalized to the untreated (DMSO 0.1%) control group. Asterisks above the error bars represent a significant difference of berzosertib treatment compared to the DMSO control group (*: p < 0.05; **p < 0.01; ***: p < 0.001; ****: p < 0.0001). Error bars represent the standard error of the mean

Apoptosis was measured using the Caspase-Glo® 3/7 assay. Cal-27 and FaDu cells were treated with berzosertib, 4 Gy of irradiation or cisplatin or a combination of two agents for 48 h (Fig. 5). In Cal-27 cells, 0.25 µM berzosertib increased apoptosis to 279%, radiation to 232% and 2.5 µM cisplatin treatment to 190% (p < 0.0001). Combination of berzosertib and irradiation significantly increased apoptosis levels to 420% (p < 0.0001). When berzosertib was combined with cisplatin, apoptosis levels were increased to 326%. Statistical analysis revealed a significant increase in apoptosis when combination treatment was compared with cisplatin monotherapy (p < 0.0001), but not in comparison with berzosertib monotherapy (p = 0.056).

In FaDu cells, 0.5 µM berzosertib treatment increased apoptosis to 244% (p < 0.0001), while treatment with radiation (123%) or 5 µM cisplatin (117%) did not increase apoptosis levels. Combination therapy, significantly enhanced induction of apoptosis to 295% after treatment with berzosertib and irradiation (p ≤ 0.007), and 434% after combination of berzosertib and cisplatin (p < 0.0001).

### Migration is partially inhibited by berzosertib

The effect of berzosertib treatment on cell migration was evaluated with a migration assay using 2-well inserts (Fig. 5). Berzosertib treatment inhibited cell migration of Cal-27 at a dose of 0.25 µM and higher. In detail, while untreated cells led to a gap closure of 98%, treatment with 0.25 µM and 0.5 µM berzosertib decreased gap closure to 82% (p = 0.011) and 50% (p < 0.0001), respectively. Migration of FaDu cells was inhibited at 0.25 µM and 0.5 µM after 24 h and 0.5 µM after 48 h. In more detail, after 24 h of incubation, untreated FaDu cells exhibited a gap closure of 41%, whereas treatment with 0.25 µM and 0.5 µM resulted in a reduced gap closure of 24% for both concentrations (p < 0.001).

## Discussion

Although treatment regimens for HNSCC have undergone some incremental improvements over the last decades, survival rates of HNSCC patients are still low [3, 23–26]. Activation of ATR is an important response mechanism to DNA damage caused by radio- and chemotherapy in HNSCC [2]. The ATR inhibitor berzosertib shows potent preclinical anticancer effects in several cancers and is well tolerated in clinical studies [5]. In HNSCC, first studies showed increased sensitivity to simultaneous treatment with berzosertib and cisplatin or irradiation [6, 27, 28]. Thus, this study aimed to further elucidate combination treatment and provide additional evidence for the use of berzosertib in combination with irradiation and cisplatin in HNSCC.

In this study, Berzosertib inhibited cell viability and had a proapoptotic effect in the investigated HNSCC cell lines with comparable IC_50_ values observed in other cancer entities [7, 29–33]. Faulhaber et al. showed a cytotoxic effect in HPV-positive and HPV-negative HNSCC cell lines and no significant effect on normal fibroblasts [6]. In clinical trials, a plasma concentration of up to 4410 ng/ml (approx. equivalent to 9.51 µmol/l) was well tolerated [11–14]. Therefore, we presume that berzosertib exhibits cytotoxicity in HNSCC cell lines at clinically relevant concentrations.

Although berzosertib as a standalone treatment appears promising [7, 29–33], combination with radiation treatment may potentiate response rates due to the common dependance of HNSCC on ATR as a damage response mechanism [2]. Faulhaber et al. demonstrated that berzosertib slightly increases radiosensitivity when combined with 2 Gy of irradiation in HNSCC cell lines [6]. However, the impact on high-dose irradiation or the mode of interaction (additive vs. synergistic) was not evaluated. The radiosensitizing effect in HNSCC is further supported by Chen et al. [28]. In this study, the combination of berzosertib and irradiation significantly decreased cell viability and led to a reduction of colony formation in an additive or synergistic manner. The addition of berzosertib to irradiation treatment increased apoptosis levels by 1.8–2.4 fold. This is especially interesting in the radioresistant cell line FaDu [34]. Here, the addition of berzosertib to irradiation potently reduced cell viability and colony formation even at low doses. In other cancer entities, berzosertib significantly enhances radiosensitivity in esophageal cancer, colorectal cancer, melanoma and lung cancer [7, 35, 36]. Furthermore, the clonogenic survival rate was reduced at similar concentrations [7–9, 31, 35]. Interestingly, there is no enhanced effect of radiation on normal fibroblasts [7, 8]. Radiation-induced DNA damage triggers cell cycle checkpoints to ensure DNA integrity. The G1/S checkpoint is commonly impaired in HNSCC cells; thus, they rely on the S and G2/M checkpoints, which are activated by ATR. Inhibition of ATR is therefore lethal in the G1/S impaired state as the DNA damage can no longer be managed. Unlike HNSCC cells, regular fibroblasts can still repair radiation-induced DNA damage through the intact G1/S checkpoint [2]. This may explain why addition of berzosertib has no radiosensitizing effect on normal fibroblasts. Based on these observations, we hypothesize that the adverse effects of irradiation on normal tissue are not increased by the addition of berzosertib.

ATR plays a pivotal role in the management of cisplatin-induced DNA damage [37–40]. Inhibition through berzosertib therefore sensitizes cells to cisplatin treatment in several cancers [28, 29, 41, 42]. The cisplatin induced DNA-damage is managed by ATR, which in turn activates p53 and other downstream targets [40, 43]. However, p53 is commonly inactivated or lost in HNSCC [2] and mutation of p53 is linked to a decreased sensitivity to cisplatin treatment [44]. Studies have shown, that loss of p53 increases dependance on ATR to manage cisplatin induced-DNA damage [40, 43]. In this study, the combination of berzosertib and cisplatin significantly inhibited cell viability compared to single agent therapy. Analysis with the combination index revealed a strong synergistic effect of combination treatment in Cal-27 cells and a synergistic to additive effect in FaDu cells. The evaluation of apoptosis levels further supported the enhanced sensitivity, as apoptosis was significantly increased by combination treatment 1.7–3.7 fold. In line with our results, the combination of berzosertib and cisplatin revealed synergistic effects in esophageal and lung cancer [29, 41]. In clinical trials, the combination of cisplatin and berzosertib was well tolerated in triple-negative breast cancer and advanced solid tumors [11, 45].

Last, the effect of berzosertib on migration was investigated to evaluate whether it may inhibit metastasis. ATR is essential for keeping the nuclear plasticity under mechanical stress and interstitial migration due to its role in the nuclear envelope [46]. According to Faulhaber et al., HNSCC cell lines’ migration was not affected at 0.1µM berzosertib. [6] In our study, berzosertib showed antimigratory effects at high doses. However, serum starvation was not tolerated by our cell lines. Thus, the observed effect may be partially attributable to an inhibition of cell viability. In other cancer cells, berzosertib inhibits cell migration in liposarcoma [47] and gastric cancer [32].

There are some limitations of this study: Berzosertib was investigated in vitro in a 2D cell culture and only in two HNSCC cell lines. The mechanistic background of berzosertib treatment on ATR inhibition and cell cycle regulation was not investigated as this was previously reported by Faulhaber et al. and Dobler et al. [6, 27].

In summary, this study underlines the cytotoxic effect of berzosertib treatment at clinically relevant doses. Combination treatment with radiation or cisplatin showed an additive or synergistic effect. Altogether, we conclude that berzosertib might be a promising agent for combination treatment with cisplatin and irradiation and could potentially help to overcome p53-associated treatment resistance in HNSCC.

## Data Availability

The datasets from this study are available from the corresponding author on reasonable request.

## Abbreviations

ATR: ataxia telangiectasia and Rad3 related
BER: Berzosertib
CI: Combination Index
CIS: Cisplatin
DMSO: Dimethyl sulfoxide
HNSCC: Head and neck squamous cell carcinoma
SEM: Standard error of the mean
ZIP: Zero Interaction Potency
95%CI: 95% confidence interval
